# Immunological Change in a Parasite-Impoverished Environment: Divergent Signals from Four Island Taxa

**DOI:** 10.1371/journal.pone.0000896

**Published:** 2007-09-19

**Authors:** Jon S. Beadell, Colm Atkins, Erin Cashion, Michelle Jonker, Robert C. Fleischer

**Affiliations:** 1 Genetics Program, National Zoological Park, Washington, D. C., United States of America; 2 Department of Biology, University of Maryland, College Park, Maryland, United States of America; 3 Australian Institute of Marine Science, Townsville, Queensland, Australia; Columbia University, United States of America

## Abstract

Dramatic declines of native Hawaiian avifauna due to the human-mediated emergence of avian malaria and pox prompted an examination of whether island taxa share a common altered immunological signature, potentially driven by reduced genetic diversity and reduced exposure to parasites. We tested this hypothesis by characterizing parasite prevalence, genetic diversity and three measures of immune response in two recently-introduced species (*Neochmia temporalis* and *Zosterops lateralis*) and two island endemics (*Acrocephalus aequinoctialis* and *A. rimitarae*) and then comparing the results to those observed in closely-related mainland counterparts. The prevalence of blood parasites was significantly lower in 3 of 4 island taxa, due in part to the absence of certain parasite lineages represented in mainland populations. Indices of genetic diversity were unchanged in the island population of *N. temporalis*; however, allelic richness was significantly lower in the island population of *Z. lateralis* while both allelic richness and heterozygosity were significantly reduced in the two island-endemic species examined. Although parasite prevalence and genetic diversity generally conformed to expectations for an island system, we did not find evidence for a pattern of uniformly altered immune responses in island taxa, even amongst endemic taxa with the longest residence times. The island population of *Z. lateralis* exhibited a significantly reduced inflammatory cell-mediated response while levels of natural antibodies remained unchanged for this and the other recently introduced island taxon. In contrast, the island endemic *A. rimitarae* exhibited a significantly increased inflammatory response as well as higher levels of natural antibodies and complement. These measures were unchanged or lower in *A. aequinoctialis*. We suggest that small differences in the pathogenic landscape and the stochastic history of mutation and genetic drift are likely to be important in shaping the unique immunological profiles of small isolated populations. Consequently, predicting the impact of introduced disease on the many other endemic faunas of the remote Pacific will remain a challenge.

## Introduction

Emerging disease in wildlife is an important force driving the decline and extinction of threatened populations [1]–[3] and may pose a threat to worldwide biodiversity [4]. Human-mediated environmental changes are often the root cause of disease emergence [5] and may have particularly dire consequences in island ecosystems. In Hawaii, for example, the introduction of non-native songbirds and the mosquito vector *Culex quinquefasciatus* has led to the emergence of avian malaria and avian poxvirus in endemic honeycreepers (Drepanididae), contributing to dramatic declines and contracting range limits of several species [6]–[9]. While host species that have been introduced to Hawaii from continental sources over the last several centuries are largely unaffected by avian malaria, endemic species may exhibit mortalities ranging as high as 100% [10], [summary in 11]. This suggests that the introduced strain of malaria is not unusually virulent; instead, it appears that at least some long-term island residents are unusually susceptible to this parasite.

High susceptibility of island endemics to infectious disease has been proposed as a component of an “island syndrome” [12], [13], which seeks to codify typical changes observed in body size [14], life history traits such as survival and fecundity [15], [16] and other features associated with insular organisms [17]. Several factors common to insular life could be driving susceptibility in island endemics. First, if parasite pressure is lower on islands, then parasites will contribute less to the selective forces that determine which components of immunity (e.g., specific or non-specific, constitutive or inducible; see [18]) are emphasized by island taxa. The protozoa, bacteria, viruses, and arthropods that are successfully transported to an island by avian colonists are typically only a subsample of those present in the source host population, and even those parasites may go extinct due to reduced transmission probabilities as the small island host population becomes established [19]. Thus, parasite richness is typically low on islands compared to the mainland [20], [21], though prevalence may vary depending on relative transmission efficiency and host densities [22]. On the remote islands of the Pacific in particular, even accounting for recent extinctions [23], bird communities are extremely depauperate and unlikely to sustain the diversity or abundance of parasites observed in large and diverse mainland host communities. Given the physiological costs associated with developing, maintaining, and using an immune system [24]–[28], in a parasite-impoverished environment, selection should favor birds that maximize fitness by allocating resources away from costly components of the immune system and perhaps towards other fitness-related traits such as reproductive effort [29]–[31], survival [32] or the expression of sexual ornaments [33], [34]. The immune components that are favored in a low parasite environment may be less efficient at overcoming challenges with novel parasites.

A second factor which may contribute to susceptibility of island fauna is the low genetic diversity typically associated with small population sizes [35]. Theory [36], [37] and observations on natural avian systems [38], [39] suggest that bottlenecks, such as those experienced upon colonization of an island, are most likely to decrease allelic diversity (due to the loss of rare alleles) while heterozygosity will decline only if the bottleneck is severe and the growth rate of the population is low. Additional diversity may be lost due to serial bottlenecks [40] if island populations, already constrained to be small by island size, are repeatedly reduced due to demographic stochasticity. This latter effect may be important in driving the differences in disease susceptibility observed in recently introduced versus endemic species. Observations in wild populations have confirmed the deleterious impacts of bottlenecks and inbreeding on immunological parameters [41]–[43] and parasite susceptibility [44]–[46]. However, drift is unlikely to affect all populations similarly and thus, the impact of inbreeding on disease susceptibility is not likely to be uniform [47].

The Hawaiian honeycreepers have become a model for understanding the susceptibility of a naïve fauna to exotic disease, but given a relative paucity of data on disease prevalence and consequences in island taxa (but see [3], [48]–[51]), the extent to which this model applies elsewhere across the globe is not obvious. For example, in contrast to Hawaii, the avifauna of American Samoa is characterized by stable native communities exhibiting relatively high prevalence of chronic infection with possibly indigenous blood parasites [52], [53]. Lack of clear parallels to the Hawaiian model may reflect Hawaii's extreme isolation or the unique susceptibility of the Drepanidine radiation to exotic disease. Alternatively, introduced pathogens may have decimated similarly susceptible species so quickly that parallel declines have gone unrecorded elsewhere in the world. In the Pacific region especially, which harbors 24% of all threatened birds species (BirdLife International 2006), Hawaii, the Galapagos [3], [49], [54] and New Zealand [55] have received the vast majority of attention, to the neglect of numerous other archipelagoes, many of which are extremely isolated and home to small populations of endemics.

To quantify immunological changes that may be common among island taxa, we characterized immunological responses in endemic and recently-introduced bird populations on remote islands of the Pacific and compared the results to closely-related taxa from mainland Australia. Because vertebrate immunity depends on a diversity of defenses of variable specificity and inducibility [18] and because successful immune defense may emphasize just a single component of those defenses [27], we characterized two components of immunity using techniques that were applicable to wild and, in some cases, vulnerable populations. As a measure of constitutive innate immunity, we assayed levels of natural antibodies and complement in plasma [56]. Natural antibodies are germ-line encoded molecules that are important in initial recognition of pathogens, circulate in the absence of specific stimulation, and may be linked to activation of the B-cell mediated production of specific acquired antibodies [57], [58]. They are also integral for initiating the action of complement, a suite of enzymes that function together to lyse foreign cells [59]. As an index of cell-mediated inflammatory immune response, we measured the delayed-type hypersensitivity response to injection with the plant-derived compound PHA. The swelling that results reflects the recruitment of lymphocytes, macrophages, basophils, and heterophils to the site of injection [59], [60]. This response, which integrates both innate and acquired components [60], is potentially important in the defense against intracellular parasites such as viruses and haemosporidia [32]. A strong response has been linked to increased probability of survival [32], [61], [62] and may be indicative of high exposure to parasites over evolutionary time [63].

If evolution on remote, parasite-impoverished islands necessarily leads to increased susceptibility to exotic parasites, then we might expect immune responses to be uniformly lower in island populations relative to their mainland counterparts. In addition, we would expect this pattern to be most evident in island endemics relative to species that have been introduced to an island only recently. Alternatively, island colonization may lead to variable upregulation or downregulation of immune components depending on the relative costs of those components in a new environment or life-history regime, their lability in the face of genetic changes, and the particular parasites with which they are challenged. For example, components of a potentially damaging inflammatory response (e.g. delayed-type hypersensitivity and complement) may be down-regulated while natural antibodies may be more useful in the context of a reduced and relatively stable parasite fauna. A previous study found no evidence for uniformly reduced immune function in island taxa and suggested that island life may instead favor increases in certain defenses, such as circulating haptoglobin, that are innate and inducible [13]. Here, we also find no evidence for uniformly low immune response in island taxa. We explore the divergent signatures of immunological change recovered in light of the genetic and parasitological context in which immunity has evolved and, through the consideration of both recently-introduced and endemic island species, we address the timescale on which immunological changes have occurred in an extremely isolated avifauna.

## Methods

### Avian System

**Figure 1:**
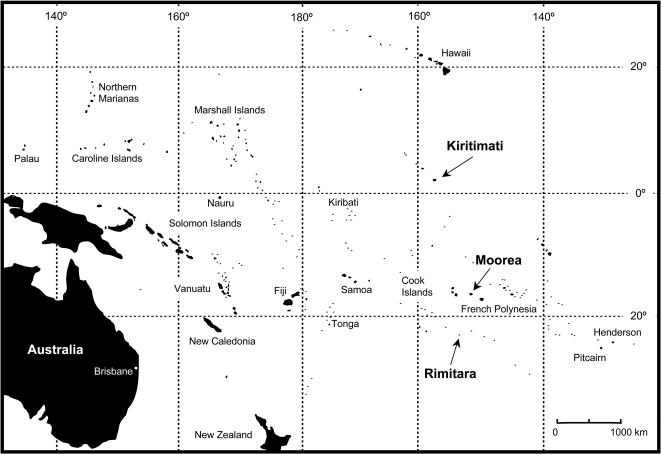
Map indicating the location of islands sampled in the central Pacific.

We characterized immune response, genetic variability, and parasite prevalence in mainland populations of three species of songbirds and compared the results to closely related island populations or species representing isolation at two different time scales. As a model of short-term isolation on islands, we sampled populations of Red-browed firetails (*Neochmia temporalis*) and Silvereyes (*Zosterops lateralis*) from their native range in Australia [64] and also from Moorea, French Polynesia (ca. 3400 km east of Fiji, 5900 km east of Australia, 6300 km southwest of Mexico; see Figure 1). *N. temporalis* was introduced to French Polynesia in the late 19^th^ century and may have been reintroduced in 1938, while *Z. lateralis* was most likely introduced in 1938 [65]. As a model of long-term evolution in an island environment, we compared two island endemic species of *Acrocephalus* reed warbler to their most closely related mainland form (*A. australis,* Fleischer et al., unpublished data). We sampled the Rimitara reed warbler (*A. rimitarae*) on Rimitara, Austral Islands, French Polynesia (ca. 3100 km east of Fiji, 5400 km east of Australia) and the Bokikokiko (*A. aequinoctialis*) on Kiritimati, Line Islands, Kiribati (ca. 6000 km northeast of Australia, 5400 km southwest of Mexico; Figure 1) as these species likely represent two distinct lineages of Pacific warblers (Fleischer et al. unpublished data) and their populations were sufficiently large and accessible to accommodate sampling. Total numbers of individuals sampled from each population are indicated in Table 1.

**Table 1:** Genetic diversity indices for introduced and endemic island taxa compared to their mainland counterpart.

Population	n	% Loci polymorphic (number screened)	Allelic richness	H_E_	H_o_	F_IS_ a
Introduced						
*Z. lateralis* (Mainland)	64	100 (12)	8.1	0.57	0.54	0.048
*Z. lateralis* (Island)	59	100 (12)	**3.8**	0.52	0.53	−0.027
*N. temporalis* (Mainland)	68	100 (6)	10.1	0.69	0.67	0.032
*N. temporalis* (Island)	34	100 (6)	8.0	0.70	0.69	0.007
Endemic						
*A. australis* (Mainland)	34	100 (12)	8.3	0.69	0.68	−0.005
*A. aequinoctialis* (Island)	25	75 (12)	**2.5**	**0.35**	**0.28**	0.124
*A. rimitarae* (Island)	56	42 (12)	**1.7**	**0.19**	**0.17**	0.145

Bold face indicates significant differences in the island index relative to the mainland (p<0.05).

a For comparative purposes, F_IS_ for *Acrocephalus* is based on only 4 loci for which gene diversity (i.e., expected heterozygosity) was non-zero in all three species

Australian populations of *N. temporalis, Z. lateralis* and *A. australis* were sampled using mistnets between June and July, 2005 at several sites in the region from Brisbane south to the Clarence River. Island populations of *N. temporalis* and *Z. lateralis* were sampled at two sites on Moorea, French Polynesia in July and August, 2005. We sampled *A. rimitarae* in August 2005 and *A. aequinoctialis* in March 2006. The timing of sampling insured that birds were not breeding, except in the case of *A. aequinoctialis* which may breed opportunistically throughout the year given evidence of terrritoriality and nesting by some individuals in both March (JSB obs.) and mid-July [66]. In this case, we did not perform immune assays on individuals that were obviously attending a nest (i.e., individuals carrying an egg or possessing a well-developed brood patch). Protocols for handling birds were approved by Animal Care and Use Committees at the University of Maryland (R-05-19) and the Smithsonian National Zoological Park (05-10).

### Parasite Screening

All captured birds were visually inspected for evidence of exposure to *Avipoxvirus* spp. (wartlike lesions on exposed skin). In addition, we screened blood smears for trypanosomes and microfilaria. For each slide, we scanned 30 fields at 100× and 50 fields at 500× magnification. Finally, we screened DNA, extracted from blood samples using DNeasy kits (Qiagen), for evidence of haematozoa in the genera *Haemoproteus, Leucocytozoon,* and *Plasmodium* using molecular methods described previously [67], [68]. PCR methods generally provide a more reliable means of detecting these haematozoa than microscopy [69]. Briefly, we used primers F2/R2 and 213F/372R to detect parasite infections. The latter includes restriction sites that are diagnostic for the three different parasite genera. In order to evaluate the diversity of parasite lineages present in any population, we used forward primers F2, Fifi, or 3760F with reverse primer 4292rw2 to amplify a 295 to 533 bp fragment of cytochrome b, which was then sequenced and compared to sequences on GenBank to confirm parasite identification. We tested for significant differences in prevalence between island and mainland populations using Fisher's exact test, since all comparisons involved cells with low values. We also included an index of infection (infected or not) as an explanatory variable in an ANCOVA testing for differences in immune response between island and mainland taxa (described below).

### Genetic Variability

We quantified levels of genetic diversity using microsatellites designed by previous authors for use with taxa related to *N. temporalis* (6 loci; [70]), *Z. lateralis* (12 loci; [71], [72]), and *Acrocephalus* spp. (12 loci; [73], [74]). Loci chosen for use across species of Pacific *Acrocephalus* were originally isolated from distantly related taxa (*A. arundinaceus* or *A. seychellensis*) and therefore, ascertainment bias should not contribute to any differences in diversity observed between species. Generally, PCR reactions were carried out in a total volume of 10 uL with 1× PCR buffer, 1 U of AmpliTaq DNA polymerase (Applied Biosystems), 0.2 mM each dNTP (NEB), 0.5 uM each primer, and optimized concentrations of MgCl and/or betaine (see Supporting Information, Table S1, for exact conditions). Products were separated on an ABI 3100 Genetic Analyzer (Applied Biosystems). Alleles were aligned and scored using Genotyper 2.5 (Perkin-Elmer) and manually binned. All loci were tested for significant deviations from Hardy-Weinberg equilibrium (HWE) in GENEPOP [75]. Samples that yielded homozygotes at any locus showing significant departures from HWE were rerun at less stringent conditions to reduce the likelihood of allelic dropout.

Values for observed heterozygosity (H_o_) were obtained from GENEPOP while values for allelic richness, gene diversity (i.e., expected heterozygosity H_E_) and the coefficient of inbreeding (F_IS_) were calculated using FSTAT v 2.9.3 [76]. Differences between mainland and island values were tested for significance across loci within each species (i.e., *N. temporalis, Z. lateralis*) or species group (i.e., *Acrocephalus* spp.) using a Wilcoxon signed-ranks test implemented with SAS v 9.1. In addition, for each individual, we calculated homozygosity by locus (HL) using microsatellite allele data and the Excel macro Cernicalin [77]. HL provides a measure of inbreeding (i.e. parental relatedness) that gives greater weight to loci with more alleles exhibiting more uniform frequencies; this approach may be less biased than other measures of inbreeding such as internal relatedness (IR; [78]). To test for a significant effect of inbreeding on immune measures, we included HL as a fixed effect in the ANCOVA described below.

### Immunological Tests

We characterized two components of the avian immune system. As a measure of investment in the cell-mediated inflammatory immune response, we challenged a subsample of captured birds with the plant-derived mitogen phytohaemagglutinin (PHA). Following the basic protocol of Smits et al. [79], we measured the patagium of captured birds to the nearest 0.01 mm with a digital small-face spline micrometer (Fowler) prior to and 24 hours after injection with PHA. Each measurement was repeated three times and averaged. Swelling reflects the recruitment of various leukocytes to the site of antigen injection as well as local inflammation of the tissue [60]. For *N. temporalis* and *Z. lateralis*, we injected 20 uL of a 1.5 mg/mL solution of PHA (Sigma L9017) in PBS buffer (Sigma P4417) into the patagium. Because early testing revealed that *A. australis* did not respond to this dosage, we injected all *Acrocephalus* spp. with 25 ul of a 3 mg/mL solution. Birds were housed in portable cages in the shade and provided with *ad libitum* water and food (seeds, fruit, or larval-stage invertebrates depending on the species).

We also characterized two measures of constitutive innate immunity in plasma sampled from island and mainland populations. In the case of the island endemics *A. aequinoctialis* and *A. rimitarae*, as well as island and mainland populations of *N. temporalis* and *Z. lateralis*, plasma was obtained from blood samples taken immediately after capture of individuals that did not undergo further immunological testing. In the case of *A. australis*, several blood samples (n = 15) were collected after completion of the PHA assay (see results). Plasma was stored in a minus 20°C freezer in the field and then transferred to a −80°C freezer until assayed (June 2006). We followed the protocol developed by Matson et al. [56] to measure levels of natural antibodies, as indicated by the agglutination of rabbit red blood cells, and levels of complement, as indicated by the lysis of these foreign cells. Assays were repeated on samples for which sufficient plasma was available and scores for these samples were averaged.

Least squares means for mainland and island immune responses were generated and tested separately for each taxa using contrasts in PROC MIXED (SAS v 9.1). We employed an ANCOVA framework with immune response as the dependent variable, population (or species in the case of *Acrocephalus*) as the main effect, and body condition (the residuals of the regression of mass on tarsus length), infection status (blood parasites only) and inbreeding (HL) as covariates. In all cases, we tested for a significant interaction of covariates with population before proceeding with a model that did not include the interaction term.

## Results

### Parasite Prevalence and Diversity

**Figure 2:**
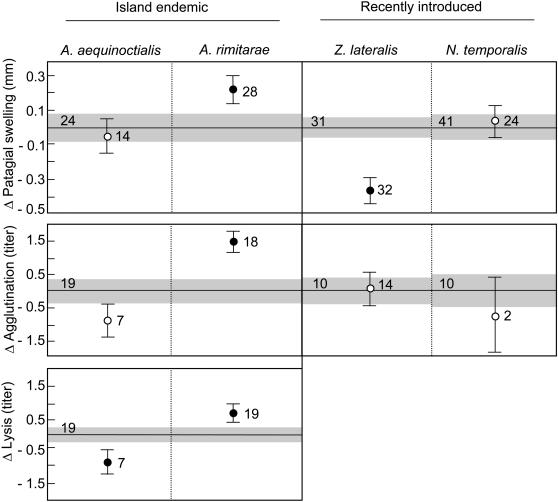
Prevalence of haematozoan parasites across mainland and island populations of *Acrocephalus* spp, *Z. lateralis*, and *N. temporalis.* Shading indicates the proportion of individuals infected with *Plasmodium* spp. (black), *Haemoproteus* spp. (light grey) and *Leucocytozoon* spp. (dark grey). Asterisks indicate significantly lower parasite prevalences in island endemic species (*Plasmodium* spp. in *A. aequinoctialis*, above, and *A. rimitarae*, below) and recently introduced island populations (*Haemoproteus* spp. in *Z. lateralis*) relative to their mainland counterparts.

We did not detect trypanosomes, microfilaria or pox lesions in any individuals and therefore, parasite analyses are limited to haematozoan infections (Figure 2). Across all individuals of the three species surveyed in Australia (n = 165), we detected 8 unique lineages of blood parasite. In contrast, we detected only a single lineage of blood parasite among the four island species (n = 174) surveyed on Kiritimati, Moorea and Rimitara. This lineage, which was only found on Moorea and was the only lineage of blood parasite recovered from any forest bird sampled there (including introduced species *N. temporalis, Z. lateralis, Pycnonotus cafer* (n = 10), *Lonchura castaneothorax* (n = 24), *Acridotheres tristis* (n = 10), *Estrilda astrild* (n = 10), *Geopelia striata* (n = 8) and the endemic dove *Ptilinopus purpuratus* (n = 5)) identically matched the strain of avian malaria introduced to Hawaii [21].

Within *Z. lateralis*, prevalence of *Haemoproteus* spp. in the introduced population on Moorea, French Polynesia (0%, n = 59) was significantly lower than that observed in Australia (39.1%, n = 64; p<0.001), where only a single lineage was detected. Prevalence of *Plasmodium* spp. did not differ significantly between island (1.7%) and mainland (6.3%) populations of *Z. lateralis*, however, mainland populations harbored at least 2 lineages of *Plasmodium*, both of which were divergent from the single lineage detected in just one individual from the French Polynesian population.

No significant differences were observed in the prevalence of any blood parasites between island (n = 34) and mainland (n = 68) populations of *N. temporalis*. We detected a single individual infected with *Leucocytozoon* spp. in Australia and this parasite was not detected in the introduced population on Moorea, French Polynesia. In addition, *Plasmodium* spp. was detected in individuals from both populations at low prevalence (1.5 to 5.9%), however the lineage in Australia was different from that found in *N. temporalis* from French Polynesia.

*Plasmodium* spp. was the only blood parasite detected in the mainland taxon *A. australis*. The prevalence of *Plasmodium* spp. in *A. australis* was 17.7% (n = 34) and we detected three distinct lineages of parasite. In contrast, we did not detect any blood parasites in the endemic reed warblers *A. aequinoctialis* (n = 25) or *A. rimitarae* (n = 56). The difference in the prevalence of *Plasmodium* spp. between island and mainland taxa was significant (p = 0.03 for both comparisons).

### Genetic diversity

No locus exhibited a significant departure from HWE within any island or mainland population when p-values were Bonferroni corrected for multiple comparisons (p>0.004 for *Acrocephalus* spp. and *Z. lateralis*; p>0.008 for *N. temporalis*). Ase13 and Ase58 exhibited significant linkage disequilibrium, but only within *A. aequinoctialis* (p<0.0001). This is likely an artifact of low diversity given that these loci have been mapped to distinct linkage groups in the related warbler *A. arundinaceus* (B. Hansson pers. comm.) and that we did not detect linkage disequilibrium between these loci in either *A. australis* or *A. rimitarae*. All other pairs of loci appeared to segregate independently within each population when p-values were corrected for multiple comparisons (p>0.0001 for *Acrocephalus* spp. and *Z. lateralis*; p>0.003 for *N. temporalis*) and therefore, we treated locus-specific indices of diversity as independent samples when comparing genetic diversity between populations.

All loci examined were polymorphic in mainland populations of *Z. lateralis* and *N. temporalis* as well as recently introduced island populations of these species. In contrast, while 100% of loci were polymorphic in the mainland species *A. australis*, 3 of 12 loci were fixed in the island endemic *A. aequinoctialis* and 7 of 12 loci were fixed in *A. rimitarae* (genetic diversity indices summarized in Table 1). Allelic richness tended to be lower in island populations of all species (or species groups) and the difference was significant in the case of the recently introduced island population of *Z. lateralis* (minimum n = 59, difference = −4.3 alleles, S = 33, p = 0.001) as well as the endemic warblers *A. aequinoctialis* (minimum n = 25, difference = −5.8 alleles, S = 39, p = 0.001) and *A. rimitarae* (minimum n = 25, difference = −6.6 alleles, S = 39, p = 0.001) relative to mainland counterparts. For *Z. lateralis* and *N. temporalis*, which were recently introduced to French Polynesia and in which allele frequency changes were unlikely to have been altered by mutation events, we tested whether low allelic diversity in French Polynesian populations could be attributed to the loss of rare alleles. For each species, we divided alleles recovered from the Australian source population into two classes depending on whether they had been retained or lost upon founding of the French Polynesian population. We excluded loci in which all alleles had been retained. For the remaining loci, we calculated the average frequency of alleles in each class and compared the difference across loci using a Wilcoxon signed-ranks test. As expected, the average frequency of alleles (in the Australian population) that were lost in French Polynesia tended to be lower than the frequency of alleles that were retained following colonization in *N. temporalis* (n = 5, Δ frequency = −0.14, S = 7.5, p = 0.063) and this difference was signficant in *Z. lateralis* (n = 11, Δ frequency = −0.226, S = 33, p = 0.001).

No significant differences were detected in either gene diversity (H_E_) or observed heterozygosity (H_o_) between recently introduced taxa and their mainland counterparts. However, both measures of heterozygosity were significantly lower in the island endemics *A. aequinoctialis* (ΔH_E_ = −0.34, S = 36, p = 0.002, ΔH_o_ = −0.42, S = 39, p = 0.001) and *A. rimitarae* (ΔH_E_ = −0.50, S = 39, p = 0.001, ΔH_o_ = −0.51, S = 39, p = 0.001) compared to *A. australis*. F_IS_, a measure of the overall level of inbreeding in a population, tended to be slightly, but not significantly, lower in recently introduced populations of *Z. lateralis* and *N. temporalis* relative to their mainland source (Table 1). Conversely, F_IS_ was higher in both island endemic warblers compared to the mainland taxon however this difference was not significant. Our power to detect a significant difference among warblers was impaired by high levels of fixation across microsatellite loci, which allowed for the comparison of F_IS_ at just 4 polymorphic loci across all three species.

### Immune Response

**Figure 3:**
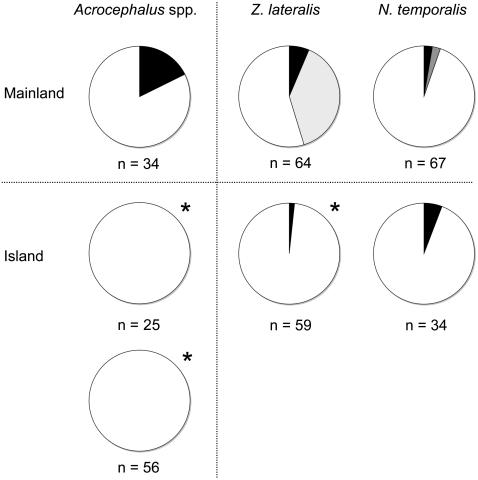
Immune response of island taxa relative to a mainland counterpart. Cell-mediated (PHA-induced patagial swelling) and constitutive innate (agglutination and lysis of rabbit red blood cells) measures of immune response in endemic and recently-introduced island taxa (circles) are indicated relative to control values (centered at zero) from a closely-related mainland taxon. Numbers indicate sample size and standard error is depicted by vertical bars, or by grey shading in the case of the mainland control. Filled circles indicate a significant difference from the mainland control.

Across all immune tests, we did not observe consistent changes in the response of island birds relative to their mainland counterparts, nor did we observe consistent trends even when island birds were grouped by island residence time (Figure 3). Among island endemics, we observed significantly greater PHA-induced swelling in *A. rimitarae* relative to its congener *A. australis* (mean difference = 0.23±0.10 mm (SE), t = 2.29, df = 60, p = 0.03), but we did not detect a similar change in *A. aequinoctialis* (diff = −0.06±0.11 mm, t = −0.55, df = 60, p = 0.58). With respect to recently introduced island taxa, the French Polynesian population of *Z. lateralis* exhibited a significant decrease in cell-mediated response relative to the Australian population (difference = −0.36±0.09 mm, t = −4.37, df = 60, p<0.001), however, we did not observe a similar difference between island and mainland populations of *N. temporalis* (diff = 0.05±0.07mm, t = 0.62, df = 62, p = 0.54).

Measures of constitutive innate immune response also yielded divergent results among comparisons of island endemics and their mainland congener. A Wilcoxon-Mann-Whitney test for differences in innate immune responses between *A. australis* that had been treated with PHA (n = 15) and those that were untreated (n = 4) revealed no significant differences (agglutination, Z = −1.18, p = 0.24; lysis, Z = −1.52, p = 0.13). Our power to detect a difference was low; however, among the 15 individuals of *A. australis* that were assayed for both cell-mediated and innate components of immunity, we found no evidence for a correlation between the extent of patagial swelling and either agglutination or lysis titers. Therefore data were combined to provide a baseline for comparison to responses in endemic warblers. Agglutination (diff = 1.5±0.42 titers, t = 3.54, df = 38, p = 0.001) and lysis (diff = 0.73±0.32 titers, t = 2.27, df = 39, p = 0.03) were both significantly higher in *A. rimitarae* relative to *A. australis*. In contrast, *A. aequinoctialis* exhibited a trend toward lower agglutination response relative to the mainland form (diff = −0.87±0.58 titers, t = −1.51, df = 38, p = 0.14), and the reduction in innate response was significant in the case of lysis (diff = −0.90±0.43 titers, t = −2.11, df = 39, p = 0.04). Across *Acrocephalus* spp., agglutination titers and lysis titers were significantly correlated (n = 46, r = 0.69, p<0.001). In recently introduced island populations, innate immune response as measured by agglutination appeared to be unchanged relative to mainland conspecifics, though our power to detect a difference among populations of *N. temporalis* was low. Neither *Z. lateralis* nor *N. temporalis* demonstrated a measurable lysis response in either island or mainland populations.

As a measure of variability in assays of innate immune components, we repeated tests of innate immune response on several samples for which sufficient plasma was available. We recovered significant correlations (p<0.001 for both) between these repeated assays for both agglutination (r = 0.80, n = 28) and lysis (r = 0.86, n = 24).

The covariates accounting for infection status and individual measure of inbreeding (HL) did not explain a significant proportion of the variance in any model of immune response in any species. The effect of body condition on immune response was significant in the models of cell-mediated (F = 7.17, df = 58, p = 0.01) and agglutination (F = 4.64, df = 19, p = 0.04) responses observed in *Z. lateralis*. Condition did not contribute significantly to any other model of immune response in either *N. temporalis* or *Acrocephalus* spp.

## Discussion

Our results do not uniformly support the hypothesis that island taxa exhibit a reduced immune response compared to mainland relatives, despite the fact that our system incorporated two key components that could theoretically drive an island syndrome: reduced exposure to pathogens and reduced genetic diversity. In addition, the divergent signals of immunological change observed in the island endemic *A. rimitarae* compared to the recently-introduced *Z. lateralis,* and also between the two endemic *Acrocephalus* spp., are not consistent with the expectation that island life uniformly emphasizes certain components of the immune system over others. With regard to levels of natural antibodies and complement, our results are similar to those of a broad multispecies study that found no uniform changes in these measures between matched island and mainland populations [13]. However, that same study revealed patterns of change in haptoglobin concentration and leukocyte composition, suggesting that additional unmeasured features of immunity may be more uniformly constrained by island life. While broader characterization of immunity, as well as wider taxon sampling, may eventually reveal patterns of change in island immune response, our results for the four island taxa sampled indicate that at least certain components of immunity may follow divergent trajectories. Studies in other island taxa suggest that these divergent patterns in immune response may be best understood in light of the particular parasite communities to which a population is exposed [80] and the particular genetic background in which immunity is evolving [46]. We examine below how these two factors may have influenced immune response at two different time scales.

### Recently-introduced island populations

In keeping with the prediction that an island syndrome may lead to reduced immune response, the island population of *Z. lateralis* exhibited a significant decrease in cell-mediated immune response relative to its mainland source population, although a measure of innate immunity was unchanged. In contrast to *Z. lateralis*, however, the immunological profile of the other recently-introduced island resident *N. temporalis* was largely similar to that found in mainland individuals. The differences in the cell-mediated immune response of these two species may be attributable to the degree to which these island invaders have escaped mainland parasites. The prevalence of haemosporidian parasites in *N. temporalis* was largely unchanged in the island population relative to the mainland. *N. temporalis* exhibited a low prevalence of infection with avian malaria (*Plasmodium* spp.) both on the mainland and in French Polynesia and the one parasite (*Leucocytozoon* spp.) that may have been lost by the island population was detected only infrequently on the mainland. In contrast, the island population of *Z. lateralis* appeared to have lost a common mainland parasite (*Haemoproteus* spp.). The absence of *Haemoproteus* spp. in the island population of *Z. lateralis* may, therefore, represent a relaxation of one of the selective forces maintaining a strong nonspecific inflammatory cell-mediated response. This potentially costly response [59] has been correlated with increased resistance to *Haemoproteus* spp. [32] and may be more relevant in the heavily-parasitized mainland population. Haemosporidia represent only one class of parasite to which island birds may be differentially exposed. However, given their potentially deleterious effects on reproduction and survival [61], [81], [82], haemosporidia are likely to be important factors structuring host immunity over evolutionary time.

Unfortunately, the population-level effects of reduced parasite exposure on immune response cannot be fully decoupled from the potential effects of reduced genetic diversity. As with blood parasite prevalence, genetic composition was similar between the immunologically indistinguishable island and mainland populations of *N. temporalis*. On the other hand, the island population of *Z. lateralis*, which exhibited lower prevalence of parasites, also exhibited reduced allelic richness. Conforming to theoretical expectation, the alleles that were lost were rare in the mainland source population. If this reduction in allelic richness at neutral loci was accompanied by a parallel loss of functional alleles (e.g., antigen-binding motifs encompassed by the major histocompatibility locus and expressed on T-cells, [83]; but see [84]), then this loss might be reflected by reduced sensitivity to a novel antigen such as PHA. Increased inbreeding has also been demonstrated to be negatively correlated to cell-mediated immune response within bottlenecked populations of song sparrows [41] and house finches [42]. In *Z. lateralis*, however, we observed similar levels of heterozygosity in island and mainland populations and found no support for a relationship between immune response and individual measures of inbreeding. Therefore, while we cannot exclude the possibility that the loss of a particular allele has contributed to a population-level effect of reduced cell-mediated immune response, inbreeding is unlikely to have affected this response.

### Island endemics

While the relative emphasis placed on particular immune components may have yet to reach a steady state in recently introduced island taxa, changes observed in the two island endemics relative to their mainland counterpart should represent long-term acclimation to their isolated environment. *A. aequinoctialis* and *A. rimitarae* exhibit average pairwise cytochrome b (cytb) divergences of 1.7% and 2.3%, respectively, from their closest mainland relative *A. australis* (based on 551 bp of cytb, K2P distances, data not shown). Using a molecular clock calibrated for passerine cytb (1.6% per million years; [85]) and correcting for ancestral polymorphism [86], [87] by subtracting the mean intraspecific divergence observed in the continental form (0.4%), these divergences equate to approximate separation times of 0.8 and 1.2 million years. Given this period of isolation, we expected that the strongest signal of an island syndrome would be found in the endemic reed warblers. Interestingly, the immunological profiles observed in *A. aequinoctialis* and *A. rimitarae* were extremely divergent and did not support this hypothesis, even though both species exhibited significant reductions in genetic diversity and reduced exposure to parasites. While *A. aequinoctialis* exhibited reduced constitutive innate immune response relative to the baseline provided by *A. australis, A. rimitarae* exhibited significantly higher innate and cell-mediated responses than the mainland control.

If investment in immunity is costly, then the latter result is particularly surprising given the degree to which *A. rimitarae* has likely been exposed to pathogens. *A. rimitarae*, like *A. aequinoctialis*, appears to have escaped the avian malaria parasites present in its mainland congener. Considering the isolation of Rimitara, the small island size (ca. 8 km^2^), and the paucity of alternative terrestrial reservoirs for pathogens (four species including junglefowl and one introduced finch), our failure to detect haematozoa in *A. rimitarae* (as well as in the co-occurring finch *Lonchura castaneothorax*, n = 10) may reflect an impoverished pathogen community in general. The same is likely true for Kiritimati, which is substantially larger (ca. 350 km^2^), but similarly isolated and host to just two additional terrestrial bird species (an introduced lorikeet and junglefowl). On the other hand, native passerines are unlikely to have escaped all of their parasites and may be exposed to at least some of the pathogens known to be present in semi-domesticated junglefowl in both French Polynesia and Kiribati (Pacific Animal Health Information System, http://www.spc.int/rahs; [49]). We surveyed only a small fraction of the total parasite community that may occur on these islands and therefore, it is possible that differential exposure to just a handful of unmeasured parasites could be driving differential immune response. Futhermore, even if parasite communities on both islands are currently impoverished, slightly different histories of parasite colonization and extinction on Rimitara and Kiritimati could be sufficient to drive differential investment in the immune system as well as differential partitioning of resources between arms of immunity.

Another possible explanation for the generally high response observed in *A. rimitarae* may be that the particular immune reponses that we measured are not extremely costly to maintain and use. While the inflammatory cell-mediated response can be costly in terms of both nutrients required [26] and potential damage inflicted to the organism itself [59], it is not well understood how these costs compare to those required to support the adaptive antibody mediated response, which we did not measure. Adaptive responses are generally cheap to use but can incur substantial developmental costs associated with the time and resources required to produce a diverse B-cell repertoire [88]. Immunocompetence in the face of a particular challenge may manifest itself by the absence of a response [89], or at least by varied emphasis on any particular arm of the immune system [27]. Therefore, our results for *A. rimitarae* would support the hypothesis of an island syndrome if the high responses observed were coupled to downregulation of a much more costly adaptive immune response. The increased cell-mediated response observed in *A. rimitarae* is consistent with the gradient of increasing inflammatory response observed in Galapagos finches exposed to fewer pathogens on increasingly smaller islands [80]. However, we did not observe a concomitant reduction in natural antibody titers, which was observed in the Galapagos finches and which may be indicative of adaptive antibody immune capacity [58]. Whatever the relative costs, the contrasting immunological profile of *A. aequinoctialis* highlights the fact that immunological signatures from endemic island taxa need not be uniform.

One factor underlying this lack of uniformity in immune response may be the stochastic nature of genetic drift acting on regulatory regions or functional genes associated with immunity. As observed in *Z. lateralis*, an initial bottleneck associated with island colonization may result in a loss of allelic diversity across the genome, which if associated with a concomitant loss of MHC diversity [74] could lead to reduced surveillance for foreign antigens. Over the longer-term, small island populations may exhibit further erosion of allelic diversity, as well as reduced heterozygosity and higher levels of inbreeding, as evidenced in *A. aequinoctialis* and *A. rimitarae*. Changes in immune response observed in bottlenecked or inbred populations [41]–[43] can be attributed to indirect effects of inbreeding, the random loss of resistance alleles, and also the loss of any advantages that may be associated with overdominance. In addition, deleterious mutations in immunologically-relevant genomic DNA can become fixed in small isolated populations given the increasing strength of drift over selection and the fact that selection may be reduced if the pathogenicity of the island environment is indeed reduced. Fixation of a mutation affecting regulation of the cell-mediated immune pathway could explain the unusually strong response observed in *A. rimitarae*. An optimal immune response is not necessarily a maximal response [90] and therefore, the strong immune response that we observed may be more indicative of a damaging allergic reaction than increased investment in that particular arm of immunity or increased ability to fight off disease.

### Conclusions

Our results indicate that while island taxa may exhibit significant changes in inflammatory cell-mediated response and in levels of innate immune compounds such as natural antibodies and complement, these immunological changes are not necessarily uniform, even among taxa with similar island residence times. This variation among immunological profiles may reflect small differences in the pathogenic landscape to which island populations are exposed and the stochastic history of mutation and genetic drift in these small populations. Unfortunately, it is not clear to what extent high or low immune response can be linked to the phenotypes in which we are most interested: disease resistance or susceptibility [91]. Similarly, while numerous studies have equated reduced genetic diversity to increased disease susceptibility [41], [42], [44], [45], [92], [93], this relationship is not universally applicable [41], [47]. Therefore, predicting the susceptibility of the many small and threatened populations of birds residing on islands of the remote Pacific may not be feasible. Even extrapolating between related species may not be warranted given the variation in susceptibility to avian malaria exhibited by the Hawaiian honeycreepers [11], all of which share a common evolutionary background. Immunity integrates not only energetic investment, which may be constrained by physiological demands of alternate life histories and differential parasite exposure, but also genetically-determined molecular recognition and regulation systems which are subject to random, population-specific effects of drift. Therefore, immunity in isolated fauna is unlikely to follow the simple heuristic of an island syndrome and may be best assayed with experimental challenges employing the particular pathogen of interest.

## Supporting Information

Table S1 PCR conditions for microsatellite loci.
(0.10 MB DOC)

## References

[1] Cooper JE (1993). Historical survey of disease in birds.. J Zoo Wildl Med.

[2] Lyles AM, Dobson AP (1993). Infectious disease and intensive management: population dynamics, threatened hosts, and their parasites.. J Zoo Wildl Med.

[3] Wikelski M, Foufopoulos J, Vargas H, Snell H (2004). Galapagos birds and diseases: invasive pathogens as threats for island species. Ecol Society 9 [online].. http://www.ecologyandsociety.org/vol19/iss1/art5.

[4] Daszak P, Cunningham AA, Hyatt AD (2000). Emerging infectious diseases of wildlife–threats to biodiversity and human health.. Science.

[5] Friend M, McLean RG, Dein FJ (2001). Disease emergence in birds: challenges for the twenty-first century.. Auk.

[6] Warner RE (1968). The role of introduced diseases in the extinction of the endemic Hawaiian avifauna.. Condor.

[7] Atkinson CT, Woods KL, Dusek RJ, Sileo LS, Iko WM (1995). Wildlife disease and conservation in Hawaii: pathogenecity of avian malaria (*Plasmodium relictum*) in experimentally infected Iiwi (*Vestiaria coccinea*).. Parasitol.

[8] Atkinson CT, Dusek RJ, Woods KL, Iko WM (2000). Pathogenecity of avian malaria in experimentally-infected Hawaii Amakihi.. J Wildl Dis.

[9] Yorinks N, Atkinson CT (2000). Effects of malaria (*Plasmodium relictum*) on activity budgets of experimentally infected juvenile Apapane (*Himatione sanguinea*).. Auk.

[10] Atkinson CT, Lease JK, Drake BM, Shema NP (2001). Pathogenicity, serological responses, and diagnosis of experimental and natural malarial infections in native Hawaiian thrushes.. Condor.

[11] Jarvi SI, Atkinson CT, Fleischer RC (2001). Immunogenetics and resistance to avian malaria in Hawaiian honeycreepers (Drepanidinae).. Stud Avian Biol.

[12] Hochberg ME, Moller AP (2001). Insularity and adaptation in coupled victim-enemy association.. J Evol Biol.

[13] Matson KD (2006). Are there differences in immune function between continental and insular birds?. Proc R Soc Lond B.

[14] Millien V (2006). Morphological evolution is accelerated among island mammals.. PLoS Biol.

[15] Wiggins DA, Moller AP, Sorensen MFL, Brand LA (1998). Island biogeography and the reproductive ecology of great tits *Parus major*.. Oecologia.

[16] Goltsman M, Kruchenkova EP, Sergeev S, Volodin I, Macdonald DW (2005). ‘Island syndrom’ in a population of Arctic foxes (*Alopex lagopus*) from Mednyi Island.. J Zool.

[17] Blondel J (2000). Evolution and ecology of birds on islands: trends and prospects.. Vie et Mileu.

[18] Schmid-Hempel P, Ebert D (2003). On the evolutionary ecology of specific immune defence.. Trends Ecol Evol.

[19] Colautti RI, Ricciardi A, Grigorovich IA, MacIsaac HJ (2004). Is invasion success explained by the enemy release hypothesis?. Ecol Lett.

[20] Fromont E, Morvilliers L, Artois M, Pontier D (2001). Parasite richness and abundance in insular and mainland feral cats: insularity or density?. Parasitol.

[21] Beadell JS, Ishtiaq F, Covas R, Melo M, Warren BH (2006). Global phylogeographic limits of Hawaii's avian malaria.. Proc R Soc Lond B.

[22] Dobson AP (1988). Restoring island ecosystems: the potential of parasites to control introduced mammals.. Conserv Biol.

[23] Steadman D (1997). The historic biogeography and community ecology of Polynesian pigeons and doves.. J Biogeogr.

[24] Klasing KC, Barnes DM (1988). Decreased amino acid requirements of growing chicks due to immunologic stress.. J Nutri.

[25] Scrimshaw NS (1991). Effect of infection on nutrient requirements.. J Parenter Enter Nutr.

[26] Lochmiller RL, Deerenberg C (2000). Trade-offs in evolutionary immunology: just what is the cost of immunity?. Oikos.

[27] Zuk M, Stoehr AM (2002). Immune defense and host life history.. Am Nat.

[28] Martin LB, Scheuerlein A, Wikelski M (2003). Immune activity elevates energy expenditure of house sparrows: a link between direct and indirect costs?. Proc R Soc Lond B.

[29] Gustafsson L, Nordling D, Andersson MS, Sheldon BC, Qvarnström A (1994). Infectious diseases, reproductive effort and the cost of reproduction in birds.. Phil Trans Roy Soc Lond B.

[30] Deerenberg C, Arpanius V, Daan S, Bos N (1997). Reproductive effort decreases antibody responsiveness.. Proc R Soc Lond B.

[31] Nordling D, Andersson M, Zohari S, Gustafsson L (1998). Reproductive effort reduces specific immune respone and parasite resistance.. Proc R Soc Lond B.

[32] Gonzalez G, Sorci G, Moller AP, Ninni P, Haussy C (1999). Immunocompetence and condition-dependent sexual advertisement in male house sparrows (*Passer domesticus*).. J Anim Ecol.

[33] Hillgarth N, Wingfield JC0, Clayton DH, Moore J (1997). Parasite-mediated sexual selection: endocrine aspects.. Host-parasite evolution.

[34] Peters A, Delhey K, Denk AG, Kempenaers B (2004). Trade-offs between immune investment and sexual signaling in male mallards.. Am Nat.

[35] Frankham R (1997). Do island populations have less genetic variation than mainland populations?. Heredity.

[36] Wright S (1931). Evolution in Mendelian populations.. Genetics.

[37] Nei M, Maruyama T, Chakraborty R (1975). The bottleneck effect and genetic variability in populations.. Evol.

[38] Baker AJ, Moeed A (1987). Rapid genetic differentiation and founder effect in colonizing populations of common mynas (*Acridotheres tristis*).. Evol.

[39] Tarr CL, Conant S, Fleischer RC (1998). Founder events and variation at microsatellite loci in an insular passerine bird, the Laysan finch (*Telespiza cantans*).. Mol Ecol.

[40] Clegg SM, Degnan SM, Kikkawa J, Moritz C, Estoup A (2002). Genetic consequences of sequential founder events by an island-colonizing bird.. Proc Natl Acad Sci U S A.

[41] Reid JM, Arcese P, Keller LF (2003). Inbreeding depresses immune response in song sparrows (*Melospiza melodia*): direct and inter-generational effects.. Proc Roy Soc Lond B.

[42] Hawley DM, Sydenstricker KV, Kollias GV, Dhondt AA (2005). Genetic diversity predicts pathogen reisistance and cell-mediated immunocompetence in house finches.. Biol Lett.

[43] Hale KA, Briskie JV (2007). Decreased immunocompetence in a severely bottlenecked population of an endemic New Zealand bird.. Anim Conserv.

[44] Acevedo-Whitehouse, Gulland F, Greig D, Amos W (2006). Inbreeding: disease susceptibility in California sea lions.. Nature.

[45] Pearman PB, Garner TWJ (2005). Susceptibility of Italian agile frog populations to an emerging strain of *Ranavirus* parallels population genetic diversity.. Ecol Lett.

[46] Whiteman NK, Matson KD, Bollmer JL, Parker PG (2006). Disease ecology in the Galapagos Hawk (*Buteo galapagoensis*): host genetic diversity, parasite load and natural antibodies.. Proc R Soc Lond B.

[47] Speilman D, Brook BW, Briscoe DA, Frankham R (2004). Does inbreeding and loss of genetic diversity decrease disease resistance?. Conserv Genet.

[48] Goltsman M, Kruchenkova EP, Macdonald DW (1996). The Mednyi Arctic foxes: treating a population imperilled by disease.. Oryx.

[49] Gottdenker NL, Walsh T, Vargas H, Merkel J, Jimenez GU (2005). Assessing the risks of introduced chickens and their pathogens to native birds in the Galapagos archipelago.. Biol Conserv.

[50] Smits JE, Tella JL, Carrete M, Serrano D, Lopez G (2005). An epizootic of avian pox in endemic short-toed larks (*Calandrella rufescens*) and Berthelot's pipits (*Anthus berthelotti*) in the Canary Islands, Spain.. Vet Pathol.

[51] Clifford DL, Mazet JAK, Dubovi EJ, Garcelon DK, Coonan TJ (2006). Pathogen exposure in endangered island fox (*Urocyon littoralis*) populations: implications for conservation management.. Biol Conserv.

[52] Jarvi SI, Farias MEM, Baker H, Freifeld HB, Baker P (2003). Detection of avian malaria (*Plasmodium* spp.) in native land birds of American Samoa.. Conserv Genet.

[53] Atkinson CT, Utzurrum RC, Seamon JO, Savage AF, LaPointe DA (2007). Hematozoa of forest birds in American Samoa–evidence for a diverse, indigenous parasite fauna from the South Pacific.. Pac Conserv Biol.

[54] Parker PG, Whiteman NK, Miller RE (2006). Conservation medicine on the Galapagos Islands: partnerships among behavioral, population, and veterinary scientists.. Auk.

[55] Tompkins DM, Gleeson DM (2006). Relationship between avian malaria distribution and an exotic invasive mosquito in New Zealand.. J R Soc New Zeal.

[56] Matson KD, Ricklefs RE, Klasing KC (2005). A hemolysis-hemagglutination assay for characterizing constitutive innate humoral immunity in wild and domestic birds.. Devel Comp Immunol.

[57] Ochsenbein AF, Zinkernagel RM (2000). Natural antibodies and complement link innate and acquired immunity.. Immunol Today.

[58] Parmentier HK, Lammers A, Hoekman JJ, Reilingh Gde V, Zaanen ITA (2004). Different levels of natural antibodies in chickens divergently selected for specific antibody responses.. Develop Comp Immunol.

[59] Janeway CA, Travers P, Walport M, Sclomchik MJ (2005). Immunobiology, 6th ed.

[60] Martin LB, Han P, Lewittes J, Kuhlman JR, Klasing KC (2006). Phytohemagglutinin-induced skin swelling in birds: histological support for a classic immunoecological technique.. Funct Ecol.

[61] Merino S, Moreno J, Sanz JJ, Arriero E (2000). Are avian blood parasites pathogenic in the wild? A medication experiment in blue tits (*Parus caeruleus*).. Proc R Soc Lond B.

[62] Moller AP, Saino N (2004). Immune response and survival.. Oikos.

[63] Martin TE, Moller AP, Merino S, Clobert J (2001). Does clutch size evolve in response to parasites and immunocompetence?. Proc Nat Acad Sci USA.

[64] Blakers M, Davies SJJF, Reilly PN (1985). The atlas of Australian birds.

[65] Long JL (1981). Introduced birds of the world.

[66] Milder SL, Schreiber RW (1982). Notes on the nesting behavior of *Acrocephalus aequinoctialis*.. Bull Brit Ornithol Council.

[67] Beadell JS, Gering E, Austin J, Dumbacher JP, Peirce M (2004). Prevalence and differential host-specificity of two avian blood parasite genera in the Australo-Papuan region.. Mol Ecol.

[68] Beadell JS, Fleischer RC (2005). A restriction enzyme-based assay for distinguishing between avian haematozoa.. J Parasitol.

[69] Richard FA, Sehgal RNM, Jones HI, Smith TB (2002). A comparative analysis of PCR-based detection methods for avian malaria.. J Parasitol.

[70] Sefc KM, Payne RB, Sorenson MD (2001). Characterization of microsatellite loci in village indigobirds *Vidua chalybeata* and cross-species amplification in estrildid and ploceid finches.. Mol Ecol Notes.

[71] Degnan SM, Robertson BC, Clegg SM, Moritz C (1999). Microsatellite primers for the study of gene flow and mating systems in white-eyes (*Zosterops*).. Mol Ecol.

[72] Frentiu FD, Lange CL, Burke T, Owens IPF (2003). Isolation of microsatellite loci in the Capricorn silvereye, *Zosterops lateralis chlorcephalus* (Aves: Zosteropidae).. Mol Ecol Notes.

[73] Hansson B, Bensch S, Hasselquist D, Lillandt B-G, Wennerberg L (2000). Increase of genetic variation over time in a recently founded population of great reed warblers (*Acrocephalus arundinaceus*) revealed by microsatellites and DNA fingerprinting.. Mol Ecol.

[74] Richardson DS, Jury FL, Dawson DA, Salgueiro P, Komdeur J (2000). Fifty Seychelles warbler (*Acrocephalus sechellensis*) microsatellite loci polymorphic in Sylviidae species and their cross-species amplification in other passerine birds.. Mol Ecol.

[75] Raymond M, Rousset F (1995). GENEPOP (version 1.2): population genetics software for exact tests of ecumenicism.. J Hered.

[76] Goudet J (1995). Fstat version 1.2: a computer program to calculate Fstatistics.. J Hered.

[77] Aparicio JM, Ortego J, Cordero PJ (2006). What should we weigh to estimate heterozygosity, alleles or loci?. Mol Ecol.

[78] Amos W, Wilmer JW, Fullard K, Burg TM, Croxall JP (2001). The influence of parental relatedness on reproductive success.. Proc R Soc Lond B.

[79] Smits JE, Bortolotti GR, Tella JL (1999). Simplifying the phytohaemagglutinin skin-testing technique in studies of avian immunocompetence.. Funct Ecol.

[80] Lindström KM, Foufopoulos J, Pärn H, Wikelski M (2004). Immunological investments reflect parasite abundance in island populations of Darwin's finches.. Proc R Soc Lond B.

[81] Bennett GF, Peirce MA, Ashford RW (1993). Avian haematozoa: mortality and pathogenicity.. J Nat Hist.

[82] Valkiunas G (2005). Avian malaria parasites and other haemosporidia..

[83] Hansson B, Richardson DS (2005). Genetic variation in two endangered *Acrocephalus* species compared to a widespread congener: estimates based on functional and random loci.. Anim Conserv.

[84] Aguilar A, Roemer G, Debenham S, Binns M, Garcelon D (2004). High MHC diversity maintaine by balancing selection in an otherwise genetically monomorphic mammal.. Proc Nat Acad Sci U S A.

[85] Fleischer RC, McIntosh CE, Tarr CL (1998). Evolution on a volcanic conveyor belt: Using phylogenetic reconstructions and K-Ar-based ages of the Hawaiian Islands to estimate molecular evolutionary rates.. Mol Ecol.

[86] Nei M, Li W-H (1979). Mathematical model for studying genetic variation in terms of restriction endonucleases.. Proc Nat Acad Sci U S A.

[87] Avise JC, Walker D, Johns GC (1998). Speciation durations and Pleistocene effects on vertebrate phylogeography.. Proc R Soc Lond B.

[88] Humphrey BD, Koutsos EA, Klasing KC, Lyons TP, Jasques KA (2002). Requirements and priorities of the immune system for nutrients.. Nutrition biotechnology in the feed and food industries: Proceedings of Alltech's 18th annual symposium.

[89] Boots M, Bowers RG (2004). The evolution of resistance through costly acquired immunity.. Proc R Soc Lond B.

[90] Viney ME, Riley EM, Buchanan KL (2005). Optimal immune responses: immunocompetence revisited.. TREE.

[91] Adamo SA (2004). How should behavioural ecologists interpret measurements of immunity?. Anim Behav.

[92] O'Brien SJ, Roelke ME, Marker L, Newman A, Winkler CA (1985). Genetic basis for species vulnerability in the cheetah.. Science.

[93] Roelke ME, Martenson JS, O'Brien SJ (1993). The consequences of demographic reduction and genetic depletion in the endangered Florida panther.. Curr Biol.

