# Supplementary material for: Immunological Change in a Parasite-Impoverished Environment: Divergent Signals from Four Island Taxa
Source: PLoS One. 2007 Sep 19;2(9):e896. doi: 10.1371/journal.pone.0000896 (PMC1975468; doi:10.1371/journal.pone.0000896)
Supplement: Table S1 — PCR conditions for microsatellite loci. (0.10 MB DOC) [file pone.0000896.s001.doc]

**Table S1. PCR conditions for microsatellite loci.**

| Taxa | Locus | Tm (°C) | Mg (mM) | Betaine (%) | BSA (mg/mL) | Cycles | Reference |
| --- | --- | --- | --- | --- | --- | --- | --- |
|  |  |  |  |  |  |  |  |
| *N. temporalis* | IND7 | 54 | 1.5 | -- | -- | 35 | [70] |
|  | IND8 | 56 | 1.0 | -- | -- | 35 | [70] |
|  | IND28 | 56 | 1.5 | -- | -- | 35 | [70] |
|  | IND29 | 56 | 1.5 | -- | -- | 35 | [70] |
|  | IND38 | 54 | 1.5 | -- | -- | 35 | [70] |
|  | IND41 | 54 | 1.5 | -- | -- | 35 | [70] |
|  |  |  |  |  |  |  |  |
| *Z. lateralis* | ZL12 | 58 | 1.5 | 10 | -- | 35 | [71] |
|  | ZL14 | 58 | 2.0 | -- | 1.0 | 35 | [71] |
|  | ZL18 | 58 | 1.5 | 10 | 1.0 | 35 | [71] |
|  | ZL22 | 57 | 1.5 | 10 | -- | 35 | [71] |
|  | ZL35 | 60 | 1.5 | 10 | -- | 35 | [71] |
|  | ZL38 | 56 | 2.0 | -- | -- | 35 | [71] |
|  | ZL41 | 53 | 2.0 | -- | 1.0 | 35 | [72] |
|  | ZL44 | 53 | 1.0 | 10 | -- | 30 | [72] |
|  | ZL45 | 58 | 1.5 | -- | -- | 39 | [72] |
|  | ZL46 | 54 | 2.0 | 10 | 1.0 | 35 | [72] |
|  | ZL50 | 59 | 1.5 | -- | -- | 35 | [72] |
|  | ZL54 | 58 | 1.5 | 10 | -- | 35 | [72] |
|  |  |  |  |  |  |  |  |
| *Acrocephalus* spp. | Aar2 | 60 | 1.5 | -- | -- | 35 | [73] |
|  | Ase7 | 60 | 1.0 | -- | -- | 35 | [74] |
|  | Ase9 | 60 | 1.0 | -- | -- | 35 | [74] |
|  | Ase11 | 62 | 1.5 | -- | -- | 35 | [74] |
|  | Ase12 | 60 | 1.5 | -- | -- | 35 | [74] |
|  | Ase13 | 62 | 1.5 | -- | -- | 35 | [74] |
|  | Ase34 | 60 | 1.5 | -- | -- | 35 | [74] |
|  | Ase48 | 60 | 2.0 | -- | -- | 35 | [74] |
|  | Ase51 | 60 | 1.5 | -- | -- | 35 | [74] |
|  | Ase56 | 60 | 1.5 | -- | -- | 35 | [74] |
|  | Ase57 | TD^a^ | 1.5 | -- | -- | 35 | [74] |
|  | Ase58 | 60 | 2.0 | -- | -- | 35 | [74] |

^a^ Touch-down cycle
